# Assessment of Metal Intake by Selected Food Supplements Based on Beehive Products

**DOI:** 10.3390/foods11091279

**Published:** 2022-04-28

**Authors:** Mario Vujić, Dražen Lušić, Jasna Bošnir, Lato L. Pezo, Željka Kuharić, Dario Lasić, Jasenka Šabarić, Lidija Barušić, Darija Vukić Lušić

**Affiliations:** 1 Directorate for Climate Activities, Ministry of Economy and Sustainable Development, 10000 Zagreb, Croatia; mariovujic@gmail.com; 2 Department of Environmental Health, Faculty of Medicine, University of Rijeka, 51000 Rijeka, Croatia; darija.vukic.lusic@medri.uniri.hr; 3 Department of Basic Medical Sciences, Faculty of Health Studies, University of Rijeka, 51000 Rijeka, Croatia; 4 Department of Environmental Protection and Health Ecology, Andrija Štampar Teaching Institute of Public Health, 10000 Zagreb, Croatia; jasna.bosnir@stampar.hr (J.B.); zeljka.kuharic@stampar.hr (Ž.K.); dario.lasic@stampar.hr (D.L.); jasenka.sabaric@stampar.hr (J.Š.); lidija.barusic@stampar.hr (L.B.); 5 University of Applied Health Sciences, 10000 Zagreb, Croatia; 6 Institute of General and Physical Chemistry, University of Belgrade, Studentski Trg 12–16, 11000 Beograd, Serbia; latopezo@yahoo.co.uk; 7 Department of Environmental Health, Teaching Institute of Public Health of Primorje-Gorski Kotar, 51000 Rijeka, Croatia

**Keywords:** toxic metals, micronutrients, food supplements, weekly intake, recommended daily dose

## Abstract

The aim of this study was to determine the quantity of particular toxic metals (Pb, Cd, As, Hg) and micronutrients (Cr, Fe, Co, Ni, Cu, Zn, Se) in the recommended daily dose of 51 food supplements based on beehive products. Samples taken from the Croatian market were submitted for the identification/quantification of studied metals and micronutrients. It was carried out by means of inductively coupled plasma mass spectrometry (ICP-MS). Eleven samples (21.57%) showed an increased concentration of total arsenic, three samples (5.88%) contained an increased concentration of total iron, and eight samples (15.68%) had an increased concentration of total nickel. Three samples (5.88%) contained an increased concentration of zinc, while one sample (1.96%) contained an increased concentration of selenium. Increased levels of certain toxic metals and micronutrients do not pose a danger to human health because the amount identified was less than what can cause toxic effects in humans. All other analysed metals and micronutrients fell within the defined literature values. Despite certain increases in particular parameters, all samples met the established toxicity criteria. This study evidenced their safety if consumed in the recommended daily dose.

## 1. Introduction

Food supplements such as vitamins and minerals, as well as various herbal preparations, honey-based products and other beehive products, have raised particular interest in human nutrition. Their most important role is attributed to enrichment of the usual diet in order to maintain health. They have been increasingly used to improve the general condition of the body, strengthen the immune system and for the replenishment of important micronutrients. The sharp rise in production and consumption has been made possible by relatively mild regulation in many countries, which classify food supplements as food rather than medicines [1]. These products, although similar in form to medicines, do not require composition and a quality check, as is the case in the field of medicines, which, before being placed on the market, must be tested for quality, safety and efficacy [2,3]. There are many factors playing a key role in the production of safe and healthy food supplements, including the safety of beehive products that compose them. Special emphasis is placed on the food safety management system. It includes the risk analysis by establishing the critical control points (HACCP) and is based on the principles described in Regulation (EC) No 852/2004 on the hygiene of foodstuffs [4]. Determination of the safety of food supplements includes basic, specific and targeted analyses defined by the European and national legislation [5]. Basic parameters analysed in the food supplements are microbiological parameters as well as the presence of toxic metals lead, cadmium and mercury [6,7]. Healthy food or food supplement is the one that is acceptable for consumption and does not contain harmful substances in quantities that could have a detrimental effect on human health, whether chronic or acute. When determining health safety, it is necessary to perform the analyses of characteristic parameters specific to a certain type of food, which change depending on the safety criteria set by the Croatian Institute of Public Health [8]. Beehive products can be found on the market either as finished products or as part of food supplements in various dosage forms such as capsules, lozenges, tablets, powders, granules, liquids and other forms prepared for consumption in measured quantities and/or in a special application modality. They are placed on the market individually or in combination [9,10]. When it comes to the safety of beehive products, it is important that Good Beekeeping Practice (GBP) is followed, as it represents the guidelines for every beekeeper during the production process. According to Food and Agriculture Organization (FAO), GBP represents a greater degree of diligence and responsibility for beekeepers through self-control and traceability [11]. The basic requirements that must be ensured by GBP can be summarized as a competent beekeeper, appropriate facilities for extracting honey and/or other beehive products, appropriate storage facilities for beekeeping equipment, utensils and honey, suitable beekeeping tools and its regular maintenance, healthy bee colonies as well as safe feed and water for the bees. The implementation of the GBP guidelines ensures the quality and safety of beehive products, encourages productivity and strengthens technological progress in beekeeping. Furthermore, it increases the consumer’s confidence, protects the primary producer from possible legal charges, ensures product competitiveness and enables access to the local and global market. In the production of food supplements based on beehive products, it is critical to take into account the quality of raw materials. Several studies have shown that they can contain hazardous ingredients such as toxic metals, pesticides, antibiotics and microorganisms, which can originate from the environment [12,13]. Food contamination with toxic metals is a global problem and represents a great concern about their safety. Toxic metals may cause a number of harmful health effects that occur due to acute or chronic poisoning. Arsenic is a carcinogen and can have a negative health impact, including various cancer types, reproductive system problems, arthrosclerosis, developmental disorders, cardiovascular problems, diabetes, anaemia and neurological effects [14]. Heavy metal ions can enter biochemical reactions by binding active sites in biomolecules. It disrupts homeostasis in human body and can lead to inhibition of enzymes, proteins, lipids as well as to DNA damage as a result of the free radical formation. Pb, Cd, As and Hg are metals that show affinity for binding to the sulfhydryl (-SH) group [15]. Lead has an inhibitory effect on enzymes required for the proper transport of Na^+^ and K^+^ and the incorporation of iron into protoporphyrin IX [16]. The latter results in inhibition of heme synthesis in haemoglobin [17]. Cadmium shows an inhibitory effect on oxidative phosphorylation, and stimulates the production of cytokines responsible for the development of inflammatory processes in human body [18]. Arsenic has been shown to have a detrimental effect on the biochemical processes of production of ATP [19], the main molecule for energy production, while the action of mercury is manifested in the inhibitory effect on the enzymes that leads to damage or complete failure of kidneys and liver [20]. A lack of micronutrients important for the production of enzymes, hormones and other substances responsible for the proper growth and development of the organism may also occur due to their excessive intake [21]. In that regard, the aim of this study was to determine the content of particular toxic metals (Pb, Cd, As and Hg) and micronutrients (Cr, Fe, Co, Ni, Cu, Zn and Se) in the food supplements based on beehive products, with the hypothesis that they are safe for human consumption. For that reason, this study also assessed their intake by consumers through the recommended daily dose of food supplements analysed, as well as calculated weekly intake. Likewise, the acquired results are paralleled to the results publicly available, based on the safety assessment related to their application. The obtained results are compared to the values of selected toxic criteria for individual elements as one of the important features of food safety.

## 2. Materials and Methods

### 2.1. Sampling

For the purposes of this study, 51 food supplements based on beehive products were sampled from the Croatian market. In order to assure the representativeness of the samples, they were carefully selected and divided into three main categories: royal jelly-based, propolis-based and honey-based food supplements, each consisting of *n* ≥ 15 samples for each category. The analysed products had in their composition a wide range of components, and the components deriving from beehive were present at different concentrations. Their quantities are not highlighted simply because honey, propolis and royal jelly were not the subjects of this study. Due to the limited number of honey-based food supplements on the market at the time of sampling, some of the selected products appear in two groups of food supplements. These products are marked with an asterisk in Table 1. Within the selected categories, the samples were divided according to the form of packaging available on the market and intended for human consumption [3]. Since there is a huge number of food supplements based on beehive products available on the market, for the purpose of selection for analysis, particular consideration was given to including, as many as possible, different types of food supplements. Food supplements in the research, all manufactured by registered producers, were randomly sampled (purchased) at the official public drugstores registered in Croatia. As the Croatian market is part of the larger European market, a significant portion of samples were of non-Croatian origin. Samples were marked with the corresponding sample code (Table 1) in order to protect the registered product labels and titles of manufacturers, a common practice in research that does not constitute the official monitoring.

### 2.2. Metals and Micronutrients Analysis

Heavy metals are metallic chemical elements that have a relatively high density (higher than 6 kg/dm^3^) and are toxic or poisonous at low concentrations. Many of them are called micronutrients (more than 20) and are considered essential to plants and animals, as well as to humans, particularly Ni, Cu, Fe and Zn. In dependency on their amount and the conditions of the environment, these elements may change their status and become toxic. Quite often, the beekeeping practice of harvesting bee products involves the use of beekeeping equipment containing metal parts (uncapping equipment, honey extractors, metal tanks etc.). Although modern beekeeping, in this part, relies on stainless steel equipment, possible use of damaged or older unprotected equipment, in combination to lower pH of the extracted matrix (i.e., honey), may allow the migration of elements into the matrix itself. Furthermore, several heavy metals (particularly As, Cd, Hg, and Pb) do not possess biological activity and, even at low concentrations, can show harmful effects [21]. Sample preparation for the analysis of metals and micronutrients was based on wet burning of the sample with 3 mL of concentrated nitric acid (HNO_3_) and 1 mL of hydrogen peroxide (H_2_O_2_) by means of microwave digestion. For sample preparation, analytical balance of 0.1 g sensitivity (Ohaus, USA, 2019) was used. Digestion was performed in the microwave oven SpeedWave 4/DAK100/4 (Berghof, Germany, 2019) at a temperature of 200 °C and a pressure of 200 bar. An inductively coupled plasma instrument with a mass detector (ICP-MS) 7900 (Agilent, USA, 2019) was used to identify and quantify the elements. The recording conditions on ICP-MS are given in Supplement Table S1, while Table S2 shows detection limits (LOD) and quantification limits (LOQ) for tested toxic metals and micronutrients. When calculating their estimated absolute amounts, values marked with less than (<) were treated as equal to (=) in order to take into account the most stringent restrictions for the intake of toxic metals and micronutrients in human body. For the calibration curve of each element, linearity of ≥0.999 was achieved.

### 2.3. Chemicals

Certified chemical compounds studied in this article were: Potassium bromide (PubChem CID: 253877), Potassium bromate (PubChem CID: 23673461), Nitric acid (PubChem CID: 944), Sodium hydroxide (PubChem CID: 14798), Hydrochloric acid (PubChem CID: 313), Internal standard (mix In, Bi, Rh, Sc), Argon (PubChem CID: 23968), Helium (PubChem CID: 23987), Hydrogen peroxide (PubChem CID: 784). The list of certified reference materials and the list of reagents used in this study were given in Tables S3a and S3b (Supplementary Material), respectively.

### 2.4. Ranking Procedure of the Beehive Products Food Supplements

The ranking procedure of the selected food supplements based on beehive products involved several criteria. These criteria were based on the extreme values of ICP data (toxic metals and micronutrients) and the maximal values of the toxic element criteria. The used criteria were: Benchmark Dose Lower Confidence Limit (BMDL); No Observed Adverse Effect Level (NOAEL); Lowest Observed Adverse Effect Level (LOAEL), and Derived No-Effect Level (DNEL).

### 2.5. Determination of Normalized Standard Scores

In order to perceive a more complex insight into the ranking of food supplements, standard scores of samples (SS) were calculated by integrating the BMDL01, NOAEL, BMDL10, BMDL05, LOAEL and DNEL toxic elements’ criteria scores, calculated for person of 70 kg body weight per week concentrations of particular toxic metals and micronutrients. The used ranking procedure was min–max, which is one of the most frequently used tools for comparison of different sample parameters. Based on the ratio of raw data and extreme values of the measurement used, the samples were ranked according to the following equation:

(1) $${\bar{x}}_{i}=\frac{\underset{i}{\max}\;x_{i}-x_{i}}{\underset{i}{\max}\;x_{i}-\underset{i}{\min}\;x_{i}},\;\forall i,\;\mathrm{in}\;\mathrm{case}\;\mathrm{of}\;“\mathrm{the}\mathrm{lower},\;\mathrm{the}\mathrm{betters}”\;\mathrm{criteria}$$

where $x_{i}$ represents the score of the toxic element criteria.

### 2.6. Statistical Analysis

Results were expressed as arithmetic mean ± standard deviation of triplicate analyses for all measurements. Normality of data sets was checked using the Shapiro–Wilk test. Homogeneity of variances was checked using Levene’s test. Statistical differences between three product groups were tested using analysis of variance (ANOVA) and post hoc Tukey’s HSD, assuming the significant difference when *p* < 0.05. Boxplots were used to visualise data distribution by quartiles. All data were statistically analysed using the STATISTICA 10.0 software package (StatSoft Inc., Tulsa, OK, USA).

## 3. Results

For the purposes of the research, samples of food supplements based on royal jelly (RG), propolis (PR) and honey (HO) were collected. The content of toxic metals and micronutrients was determined by the ICP-MS technique. The obtained results are shown in Table 2.

**Figure 1:**
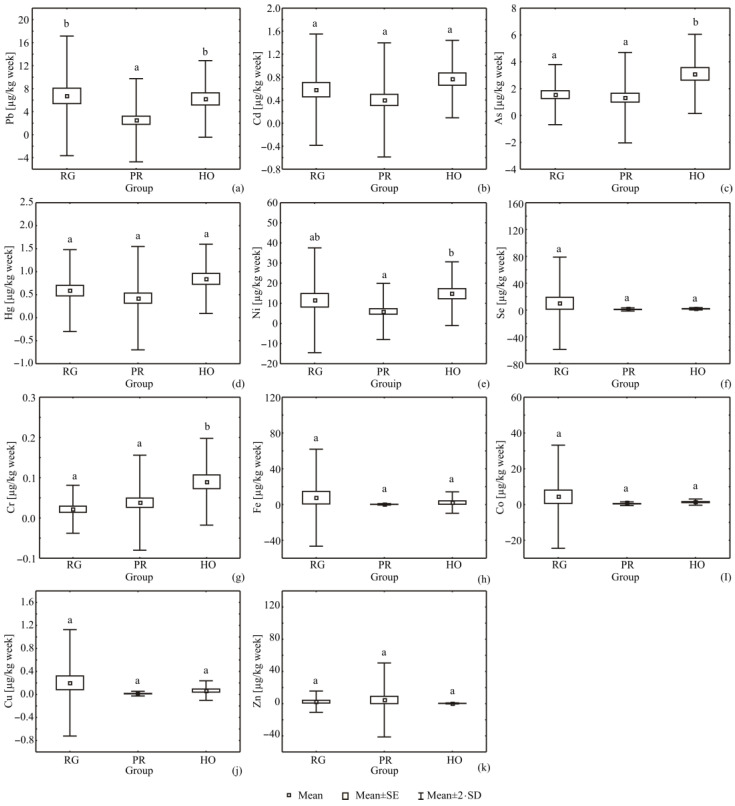
Boxplot distribution of toxic metals and micronutrients in the tested food supplements, for: (**a**) Pb, (**b**) Cd, (**c**) As, (**d**) Hg, (**e**) Ni, (**f**) Se, (**g**) Cr, (**h**) Fe, (**i**) Co, (**j**) Cu and (**k**) Zn content. Values presented in bars with the different letters are statistically different (*p* < 0.05) by means of Tukey’s HSD test. Letters are same with the subfigure’s title.

When considering the weekly intake, the results showed statistically significant differences (*p* < 0.05) in Pb content between the PR group of samples (2.51 ± 3.62 µg/kg per week) and the RG and HO group of samples (6.75 ± 5.20 and 6.22 ± 3.33 µg/kg per week, respectively) as shown in Figure 1. When considering the methodology of propolis production, it is known that propolis is obtained by purification of the raw propolis, which can contain impurities and, therefore, contamination compounds [13,22]. Moreover, the statistically significant differences were observed in As content between the RG and PR (1.56 ± 1.12 and 1.33 ± 1.68 µg/kg per week, respectively) and HO samples (3.10 ± 1.48 µg/kg per week). Significant differences were noticed for Ni content, too, between the PR (5.98 ± 6.99 µg/kg per week) and HO group (14.79 ± 7.92 µg/kg per week). Cr content also displayed significant differences between RG and PR (0.02 ± 0.03 and 0.04 ± 0.06 µg/kg per week, respectively) and the HO group of samples (0.09 ± 0.05 µg/kg per week), while no statistically significant differences were noticed for Cd, Hg, Se, Fe, Co, Cu and Zn content between all three investigated group of samples (*p* > 0.05).

According to the correlation analysis (Table 3), the positive correlation between Pb, Cd, As, Hg and Ni content was witnessed, statistically significant at *p* < 0.001 level. The positive correlation between Cr content in samples and the contents of Pb, Cd, As, Hg and Ni was statistically significant at *p* < 0.001 level. The positive correlation between Se and Cu contents showed statistical significance at a *p* < 0.001 level. According to the best authors’ knowledge and the available literature resources, until now, there were no published data regarding the connections and correlation between toxic metals and micronutrients in food supplements based on beehive products.

For the calculation of the data shown in Table 4, the amounts of toxic metals and micronutrients obtained by the analysis (listed in Table 2) and their amount expressed as the recommended daily dose were taken into account. Table 4 shows the calculated amounts of toxic metals and micronutrients in the recommended daily dose of the product, which is converted into the weekly intake. The obtained data are shown and performed as the basis of other calculations in this study. When considering the recommended daily doses of each of the analysed samples of food supplements, the calculation of the intake of particular (measured) toxic metals and micronutrients in the human body is given. Moreover, the weekly intake of toxic metals and micronutrients was calculated, too. Its values were compared with the recommended daily, weekly, and monthly intakes published in the available literature and converted into weekly intakes for normalization and easier presentation of results (Table 4). They differ for different samples and depend on the form in which the food supplement appears (drops, ampules, lozenges etc.).

In order to obtain more detailed insight and to perform a ranking procedure of the selected food supplements based on beehive products, several criteria were evaluated. For that purpose, a chemometric approach was used by integrating the extreme values of ICP data and the maximums of the toxic element criteria values as listed in Table 5. Toxic criteria maximums listed in Table 5 are derived by taking into account the value for each criterion present and provided by the European Food Safety Authority (EFSA) as well as the European Chemicals Agency (ECHA). A list of the toxic element criteria for Pb, As, Hg, Cr (VI), Co, Cd, Ni, Cu, Zn, and Se are given in Table S4 (Supplementary material) taken from the OpenFoodTox, the chemical hazards database of EFSA, as well from ECHA database [23,24,25,26,27,28,29].

Min–max normalization is a broadly used technique for the comparison of different parameters of the complex samples, particularly where the samples are ranked based on the extreme measured values of parameters [30]. The overall results of the several ranking procedures of the observed samples are given in Table 6.

## 4. Discussion

The maximum permitted amounts of certain metals that may be contained in food supplements are regulated by the Commission Regulation (EC) No. 1881/2006, setting maximum levels for certain contaminants in foodstuffs in Section 3—Metals of respective Regulation [6]. Pursuant to the above-mentioned regulation, the category “Food supplements” stipulates that the maximum permitted amount for lead is 3.0, for cadmium 3.0 and for mercury 0.1 mg/kg. Products that contain higher amounts of metals than allowed are considered unsafe for health and cannot be placed on the market. ICP-MS technique applied to analysed samples revealed that the amount of lead ranged from 0.02 to 0.275 mg/kg (FS-01 to FS-51). Determined concentrations of cadmium were found within <0.006 and 0.095 mg/kg, while the mercury values ranged from <0.007 to 0.012 mg/kg. The obtained values of all analysed metals were found below the maximum permitted amounts, indicating that analysed samples were acceptable and are considered safe for consumption (Table 2). For estimation of the safety degree of food supplements, it is important to assess the weekly intake of selected elements by consumers, taking into account the recommended daily dose (Table 4). For the purposes of this study, the weekly intake of toxic metals and micronutrients was calculated, and the obtained results were compared to the values of Provisional Tolerable Weekly Intake (PTWI), Provisional Maximum Tolerable Weekly Intake (PMTWI), Maximum Tolerable Weekly Intake (MTWI) and Tolerable Weekly Intake (TWI) available in the scientific literature for individual elements. The amount of Pb set by the recommended daily dose of the product, calculated on weekly intake, ranged from 0.22 to 20.16 µg per week, which does not exceed PTWI, which is 25 µg/kg body weight per week [23]. In terms of total Cd, all samples having Cd within the recommended daily dose of the product calculated on weekly intake (0.02 to 1.75 µg/kg body weight per week) did not exceed the TWI of 2.5 µg/kg body weight per week [23]. In the tested samples, according to the recommended daily dose of the product calculated on weekly intake, As was present in the amount of 0.04 to 7.43 µg/kg per week. An increase in As concentration, when compared to PTWI of 3.0 µg/kg body weight per week, was recorded in eleven samples (21.57%) [31]. In this particular case, it is the total arsenic where the concentration of the organic, less toxic form was found to be higher than the toxic inorganic one. These results also point out that there is a need to prescribe the maximum level of As in food and food supplements as it has been performed by Commission Regulation (EC) No. 1881/2006 for Pb, Cd and Hg [6]. In tested samples, inorganic Hg expressed as Hg in the recommended daily dose of the product calculated on weekly intake was present in the amount ranging from 0.02 to 2.04 µg/kg per week, which is less than the TWI of 4 µg/kg body weight per week [23]. Taking into account the Cr analysed in samples, Cr was present within the recommended daily dose of the product calculated on weekly intake in the amounts between 0.001 and 0.270 mg/kg per week, therefore less than PTWI, which is 0.7 mg/kg body weight per week [31]. In tested samples, the total Fe in the recommended dose of the product calculated on weekly intake was present in the amount ranging from 0.005 to 105.525 mg/kg per week. Three samples (5.88%) showed an increased concentration of total iron when compared to PMTWI, which is 5.6 mg/kg body weight (FS-04, FS-12, and FS-44). It was calculated from the provisional maximum tolerable daily intake (PMTDI) which is 0.8 mg/kg body weight per day [23]. In two of this three products (FS-12 and FS-44), the increased concentration of iron is the result of the fact that they are intended as iron supplement for people who suffer from a significant iron deficiency or anaemia and therefore are considered to have no harmful effects on human health. Tested samples showed that Co in the recommended daily dose of the product calculated on weekly intake is present in the amount from 0.01 to 56.42 µg/kg per week. It is many times lower than the MTWI of 700 µg/kg body weight per week, which was calculated from the MTDI of 100 µg/kg body weight per day [32]. Analysis of the samples showed that total Ni in the recommended daily dose of the product calculated on weekly intake was present in the amount of 0.31–52.15 µg/kg per week. Eight samples (15.68%) showed an increased concentration of total nickel when compared to TWI. It showed 19.6 µg/kg body weight per week which was calculated from the tolerable daily intake (TDI) of 2.8 µg/kg per day [23]. The results of the analysis showed that total Cu in the recommended daily dose of the product calculated on weekly intake was present in the amount from 0.0010–1.5680 mg/kg per week, many times less than PMTWI. The PTWI was 3.5 mg/kg body weight per week when calculated from PMTDI, which is 0.5 mg/kg body weight per day [33]. In the tested samples, Zn in the recommended daily dose of the product calculated on weekly intake was found in the amount ranging between 0.003 and 117.250 mg/kg per week. Three samples (5.88%) showed an increased zinc concentration when matched to PMTWI, which is 7 mg/kg body weight per week. It was calculated from PMTDI that set the Zn value at 1 mg/kg body weight per day [33]. Analysis showed that Se in the recommended daily dose of the product calculated on weekly intake was present in the amount from 0.05 to 134.40 µg/kg per week. In one sample (1.96%), there was an increased concentration of Se compared to PTWI (66 µg/kg body weight per week) [34]. Altogether it has to be emphasized that there is no existence of a particular restriction on food supplement use in current legislation, although one of the mandatory statements on such products is that the recommended Daily Allowances should not be exceeded [9,10]. It suggests that consumers should be very careful when using them, pointing to the fact that additional attention should be given to the content of food supplement labelling. One of the key aspects of food supplements safety is the establishment of the system of nutrivigilance. It is a health control system with the aim of increasing consumer safety. This can be achieved by the rapid identification of potentially harmful side effects associated with the consumption of food supplements, foods or beverages enriched with substances for nutritional and physiological needs, novel foods or ingredients of novel food and products intended for a particular group of people. French Agency for Food Environmental and Occupational Health and Safety (ANSES) developed such system of nutrivigilance for France [35,36]. In addition to France, Italy, Czech Republic, Slovenia, Ireland and Sweden are investigating, or have already begun to establish nutrivigilance at the national levels. In their study of 2019, Morgovan et al. [37] also pointed out the need of introducing the nutrivigilance system globally, with the special reference to the Romanian market. Croatian national legislation exerts the Ordinance on substances that can be added to food and used in food production and substances whose use in food is prohibited or restricted by the Croatian Ministry of Health [38]. It prescribes that propolis, royal jelly and pollen are allowed in food supplements, but do not prescribe the maximum daily allowances, as well as the additional warnings/restrictions. On the other hand, there are studies dealing with the occurrence of contact dermatitis, as well as other side effects reported by beekeepers and the consumers of beehive products [39,40]. Previously mentioned restrictions apply only to certain medicinal plants that are being used as food supplements. In addition to beehive products, other ingredients in the analysed samples, such as added parts of particular plant species, may have also contributed to the mineral composition of the product. On the other hand, it is to point out that overall quantitative analysis of the composition of studied food supplements based on the beehive products was not the topic of this study. For this reason, it was not taken into account. That is why it was not discussed how much the other ingredients may have affected or contributed to the mineral composition of the product. Although there are researches dealing with the analysis of metal content in food supplements, this is the first study of its kind that covers elements and relates them to food supplements based on beehive products. As it can be seen in the Table 6, the metal ranks are based upon the polarity marks (Table 3), which decide the sign of the rank. If the mark is “+” than the “higher the better” criteria is applied. Standard scores (SS) were calculated as the average values of all investigated metal concentration ranks, giving a specific insight in the quality of the sample, in contrast to other samples. According to this criteria, the best sample was FS-04, with a 0.658 score (between 0 and 1). The ranking criteria of the toxic element were calculated using the min–max normalization procedure, with minimum values of ICP data from Table 4, whereas the maximums were taken from Table 5. According to BMDL01 criteria, the best sample was FS-30, with a 0.944 score. Sample FS-06 was the best sample according to several criteria: BMDL10, BMDL05 and LOAEL, while sample FS-33 was the best sample according to LOAEL and DNEL criteria (Table 6).

## 5. Conclusions

Taking into consideration all selected toxicological criteria, all analysed samples of food supplements based on beehive products proved safe for human consumption. If used in the manner and in amounts prescribed by the manufacturers. In that regard, the Croatian market for such particular products shows similar features to the broader EU market it belongs to. It is important to point out that the national surveillance (inspection) system exists; nonetheless, current controls can be considered as not fully sufficient. For that reason, the results of this study may provide valuable information for national authorities responsible for the sector. That is particularly important since there can be found the overlaps in the competencies of state administration bodies responsible for food safety and food supplements as well as those responsible for approving food supplements. In that regard, the official control system should be steered in the direction of making it more applicable in practice. A somewhat superior nutrivigilance system, based on similar studies to this one, if developed, would significantly improve the existing system, which should be supported by further research in this specific area. It is also important to emphasize the need for harmonization of the legislative framework of EU countries. It is particularly important when it comes to the products containing different plant species, vitamins and minerals in quantities significantly higher than the maximum allowed, and all for the purpose of protecting the health and safety of consumers.

## Figures and Tables

**Table 1:** Food supplements used in the study.

Royal Jelly-Based Food Supplements		
Food Supplement Form	Descriptive Product Name	Sample Code
Ampoules	* Ampoules with royal jelly and honey	FS-01
	Ampoules with royal jelly for children	FS-02
	Ampoules with royal jelly	FS-03
	Royal jelly ampoules	FS-04
	Royal jelly ampoules for children	FS-05
Capsules	Capsules with royal jelly	FS-06
Pastilles	Royal jelly pastilles with vitamin C	FS-07
	Royal jelly pastilles with sweeteners	FS-08
	Royal jelly pastilles	FS-09
Syrups	Syrup with honey, royal jelly and propolis	FS-10
	Food supplement based on propolis, honey and royal jelly	FS-11
	Syrup with royal jelly and honey	FS-14
Liquid food supplements	Liquid food supplement with royal jelly and vitamin C	FS-12
	Liquid food supplement with royal jelly	FS-13
Other forms	* Product with honey and supplements	FS-15
**Propolis-Based Food Supplements**		
**Food Supplement Form**	**Descriptive Product Name**	**Sample Code**
Drops	Propolis drops	FS-16
	Propolis drops	FS-17
	Propolis drops 15%	FS-18
	Non-alcoholic propolis drops	FS-19
	Propolis drops	FS-20
	Non-alcoholic drops with propolis and echina cea	FS-21
Capsules	Capsules with propolis	FS-22
Pastilles	Propolis pastilles	FS-23
	Pastilles with propolis, vitamin C and zinc	FS-24
	Pastilles with propolis and vitamin C	FS-25
Syrups	Syrup with propolis, honey and myrtle	FS-26
	* Syrup with honey, propolis and wild thyme	FS-27
	* Syrup with propolis, honey, marshmallow, thyme and vitamins	FS-28
Sprays	Propolis spray	FS-29
	Propolis spray for children	FS-30
	Propolis water spray with vitamin B3	FS-31
	Propolis solution in spray	FS-32
	Non-alcoholic propolis solution with peppermint in spray	FS-33
Tablets	Peppermint-flavoured chewable tablets	FS-34
	Honey-flavoured chewable tablets	FS-35
	Candies with propolis and vitamins A and C	FS-36
	Chewable tablets for children, with sweetener, with the addition of vitamin C	FS-37
	Effervescent tablets with propolis and vitamin C	FS-38
Liquid food supplements	Liquid food supplement for children and adults, with marshmallow and propolis	FS-39
	Liquid food supplement with propolis	FS-40
	* Liquid food supplement with thyme and propolis	FS-41
**Honey-Based Food Supplements**		
**Food Supplement Form**	**Descriptive Product Name**	**Sample Code**
Ampoules	* Ampoules with royal jelly and honey	FS-01
Pastilles	Pastilles with sage, chamomile and honey	FS-42
Syrups	Syrup with honey, propolis and marshmallow	FS-43
	Syrup with honey, iron and vitamin C	FS-44
	Syrup for children with propolis, honey and supplements	FS-45
	* Syrup with honey, propolis and wild thyme	FS-27
	*Syrup with propolis, honey, marshmallow, thyme and vitamins	FS-28
	Syrup with honey and supplements	FS-46
Liquid food supplements	Liquid food supplement with honey and thyme	FS-47
	* Liquid food supplement with thyme and propolis	FS-41
Other forms honey with supplements	Honey with propolis and thyme	FS-48
	Product with honey, propolis, sage, wild thyme, lungwort and nettle	FS-49
	* Product with honey and supplements	FS-15
	Honey with supplements	FS-50
	Honey-based product with lemon balm and valerian	FS-51

* Products found in the two main categories of food supplements in the study.

**Table 2:** Estimated absolute concentration [mg/kg] of toxic metals and micronutrients in the tested food supplements.

Sample Code	Pb	Cd	As	Hg	Cr	Fe	Co	Ni	Cu	Zn	Se
FS-01	0.128	<0.006 *	0.017	0.007	0.117	17.800	0.005	0.305	0.470	12.700	<0.015 *
FS-02	0.176	<0.006 *	0.029	<0.007 *	0.115	1.4700	0.005	0.745	0.136	1.870	<0.015 *
FS-03	0.057	<0.006 *	0.028	<0.007 *	0.091	1.460	0.003	0.117	0.362	2.630	<0.015 *
FS-04	0.074	<0.006 *	0.017	0.007	0.200	82.100	0.014	0.093	22.400	3.070	1.920
FS-05	0.162	0.010	0.015	0.012	0.077	1.300	0.006	0.141	15.200	2.270	<0.015 *
FS-06	0.161	<0.006 *	0.016	<0.007 *	0.258	11.100	0.005	0.119	4.930	22.100	0.047
FS-07	0.143	0.007	0.018	<0.007 *	0.193	9.230	0.004	0.102	2.290	13.200	0.026
FS-08	0.118	<0.006 *	0.018	<0.007 *	0.215	16.400	0.009	0.122	0.262	5.480	<0.015 *
FS-09	0.060	<0.006 *	0.020	<0.007 *	0.135	3.830	0.004	0.091	1.190	6.340	0.021
FS-10	0.096	0.007	0.015	<0.007 *	0.071	1.980	0.009	0.101	0.180	41.40	<0.015 *
FS-11	0.086	0.006	0.019	<0.007 *	0.118	1.480	0.007	0.052	0.146	0.8860	<0.015 *
FS-12	0.093	<0.006 *	0.015	<0.007 *	0.155	1005.000	0.014	0.070	0.085	2.190	<0.015 *
FS-13	0.049	<0.006 *	0.018	0.009	0.523	2.630	0.403	0.082	0.765	179.000	<0.015 *
FS-14	0.036	<0.006 *	0.022	<0.007 *	0.528	2.530	0.006	0.071	0.328	1.890	0.020
FS-15	0.067	0.023	0.036	<0.007 *	0.966	6.120	0.021	0.185	0.845	10.600	0.017
FS-16	0.063	<0.006 *	0.047	<0.007 *	0.962	13.000	0.010	0.190	0.320	0.990	<0.015 *
FS-17	0.063	<0.006 *	0.080	<0.007 *	0.967	3.050	0.021	0.123	0.292	2.360	<0.015 *
FS-18	0.095	<0.006 *	0.065	<0.007 *	0.983	3.430	0.015	0.195	1.03	5.820	<0.015
FS-19	0.038	<0.006 *	0.019	<0.007 *	0.502	1.580	0.009	0.056	0.103	3.950	<0.015 *
FS-20	0.066	<0.006 *	0.030	<0.007 *	1.000	1.880	0.005	0.119	0.309	2.760	<0.015 *
FS-21	0.027	<0.006 *	0.015	<0.007 *	0.503	0.960	0.002	0.054	0.139	0.300	<0.015 *
FS-22	0.275	0.095	0.053	<0.007 *	0.846	168.000	0.048	0.535	7.320	82.900	0.027
FS-23	0.037	<0.006 *	0.019	<0.007 *	0.527	2.900	0.004	0.063	0.248	0.384	<0.015 *
FS-24	0.036	<0.006 *	0.015	<0.007 *	0.512	1.450	0.003	0.062	0.100	1340.000	<0.015 *
FS-25	0.031	<0.006 *	0.015	<0.007 *	0.510	0.878	0.002	0.056	0.109	0.439	<0.015 *
FS-26	0.020	<0.006 *	0.016	<0.007 *	0.507	2.010	0.012	0.063	0.109	0.394	<0.015 *
FS-27	0.035	<0.006 *	0.016	<0.007 *	0.514	1.390	0.005	0.085	0.189	1.860	<0.015 *
FS-28	0.032	<0.006 *	0.018	<0.007 *	0.511	3.510	0.006	0.073	0.179	0.954	<0.015 *
FS-29	0.068	<0.006 *	0.021	<0.007 *	0.548	2.740	0.019	0.244	0.230	0.568	<0.015
FS-30	0.056	<0.006 *	0.026	<0.007 *	0.492	1.230	0.016	0.176	0.213	0.673	<0.015 *
FS-31	0.050	<0.006 *	0.022	<0.007 *	0.637	6.630	0.007	0.110	0.122	0.492	<0.015 *
FS-32	0.069	<0.006 *	0.038	<0.007 *	0.987	2.670	0.011	0.109	0.230	1.380	<0.015 *
FS-33	0.075	<0.006 *	0.022	<0.007 *	0.538	1.800	0.003	0.098	0.109	0.636	<0.015 *
FS-34	0.040	<0.006 *	0.073	<0.007 *	0.667	21.400	0.065	0.735	0.334	1.260	0.022
FS-35	0.048	<0.006 *	0.077	<0.007 *	0.561	20.900	0.070	0.629	0.242	2.470	<0.015 *
FS-36	0.061	<0.006 *	0.017	<0.007 *	0.507	2.380	0.003	0.070	0.114	0.305	<0.015 *
FS-37	0.045	<0.006 *	0.023	<0.007 *	0.554	2.880	0.008	0.185	0.215	1.060	<0.015 *
FS-38	0.020	<0.006 *	0.017	<0.007 *	0.422	0.870	0.003	0.072	0.112	0.334	<0.015 *
FS-39	0.026	<0.006 *	0.019	<0.007 *	0.515	1.380	0.007	0.067	0.137	0.501	<0.015 *
FS-40	0.027	<0.006 *	0.014	<0.007	0.494	1.690	0.006	0.050	0.193	0.672	<0.015 *
FS-41	0.067	<0.006*	0.030	<0.007 *	1.090	2.290	0.005	0.106	0.207	1.040	<0.015 *
FS-42	0.035	<0.006 *	0.021	<0.007 *	0.556	1.290	0.006	0.087	0.162	0.605	<0.015 *
FS-43	0.036	<0.006 *	0.020	<0.007 *	0.515	1.690	0.012	0.088	0.250	1.920	<0.015 *
FS-44	0.047	<0.006 *	0.020	<0.007 *	0.560	275.000	0.024	0.109	0.367	1.120	<0.015 *
FS-45	0.024	<0.006 *	0.020	<0.007 *	0.569	0.667	0.002	0.054	0.094	0.198	<0.015 *
FS-46	0.055	<0.006 *	0.031	<0.007 *	1.030	1.360	0.006	0.101	0.193	0.761	<0.015 *
FS-47	0.044	<0.006 *	0.034	<0.007 *	0.941	1.250	0.006	0.104	0.185	0.666	<0.015 *
FS-48	0.059	<0.006 *	0.027	<0.007 *	0.971	2.170	0.009	0.121	0.283	2.010	0.016
FS-49	0.062	<0.006 *	0.037	<0.007 *	1.020	2.820	0.010	0.130	0.297	4.250	0.020
FS-50	0.085	0.010	0.017	<0.007 *	0.565	6.160	0.016	0.197	1.610	7.290	<0.015 *
FS-51	0.064	<0.006 *	0.030	<0.007 *	0.568	8.040	0.025	0.243	2.560	8.300	<0.015 *

The results were expressed as the mean value of three measurements [mg/kg]; * less than the limit of quantification of the method (LOQ).

**Table 3:** Correlation matrix of toxic metals and micronutrients in the tested food supplements.

	Cd	As	Hg	Ni	Se	Cr	Fe	Co	Cu
Pb	0.753 ^+^	0.729 ^+^	0.785 ^+^	0.764 ^+^	0.068	0.558 ^+^	0.169	0.128	0.206
Cd		0.814 ^+^	0.883 ^+^	0.586 ^+^	0.021	0.732 ^+^	0.035	0.155	0.085
As			0.886 ^+^	0.672 ^+^	0.006	0.931 ^+^	−0.014	0.130	−0.005
Hg				0.601 ^+^	0.041	0.803 ^+^	0.053	0.254	0.096
Ni					−0.005	0.498 ^+^	−0.025	0.085	0.050
Se						−0.026	0.031	−0.004	0.815 ^+^
Cr							−0.068	0.124	−0.059
Fe								−0.005	−0.001
Co									0.026

^+^ Correlation was statistically significant at *p* < 0.001 level; unmarked correlation was not statistically significant.

**Table 4:** Calculated weekly intake of toxic metals and micronutrient by average consumers for tested food supplements in view of the recommended daily dose. Pb, Cd, As, Hg, Ni, Co and Se are expressed in µg per week while Cr, Fe, Cu and Zn were expressed in mg per week.

Sample Code	Pb	Cd	As	Hg	Ni	Co	Se	Cr	Fe	Cu	Zn	Recommended Daily Dose **
FS-01	8.96	0.42	1.19	0.49	21.35	0.35	1.05	0.008	1.246	0.0329	0.889	1 ampoule = 10 mL ≈ 10 g
FS-02	12.32	0.42	2.03	0.49	52.15	0.35	1.05	0.008	0.103	0.0095	0.131	1 ampoule = 9 mL = 10 g
FS-03	3.99	0.42	1.96	0.49	8.19	0.21	1.05	0.006	0.102	0.0253	0.184	1 ampoule = 9 mL = 10 g
FS-04	5.18	0.42	1.19	0.49	6.51	0.98	134.40	0.014	5.747	1.5680	0.215	1 ampoule = 10 mL = 10 g
FS-05	11.34	0.70	1.05	0.84	9.87	0.42	1.05	0.005	0.091	1.0640	0.159	1 ampoule = 10 mL ≈ 10 g
FS-06	0.42	0.02	0.04	0.02	0.31	0.01	0.12	0.001	0.029	0.0128	0.057	1 capsule = 0.37 g
FS-07	1.24	0.06	0.16	0.06	0.89	0.03	0.23	0.002	0.080	0.0199	0.115	1 pastille = 1.24 g
FS-08	2.89	0.15	0.44	0.17	2.99	0.22	0.37	0.005	0.402	0.0064	0.134	4 pastilles = 3.50 g
FS-09	1.05	0.11	0.35	0.12	1.59	0.07	0.37	0.002	0.067	0.0208	0.111	2 pastilles = 2.50 g
FS-10	20.16	1.47	3.15	1.47	21.21	1.89	3.15	0.015	0.416	0.0378	8.694	30 mL ≈ 30 g
FS-11	6.02	0.42	1.33	0.49	3.64	0.49	1.05	0.008	0.104	0.0102	0.062	2 teaspoons = 10 mL ≈ 10 g
FS-12	9.77	0.63	1.58	0.74	7.35	1.47	1.58	0.016	105.525	0.0089	0.230	15 mL ≈ 15 g
FS-13	6.86	0.84	2.52	1.26	11.48	56.42	2.10	0.073	0.368	0.1071	25.060	20 mL ≈ 20 g
FS-14	6.30	1.05	3.85	1.23	12.43	1.05	3.50	0.092	0.443	0.0574	0.331	25 mL ≈ 25 g
FS-15	4.69	1.61	2.52	0.49	12.95	1.47	1.19	0.068	0.428	0.0592	0.742	2 teaspoons = 10 g
FS-16	0.60	0.06	0.44	0.07	1.80	0.09	0.14	0.009	0.123	0.0030	0.009	45 drops ≈ 1.35 mL ≈ 1.35 g
FS-17	0.79	0.08	1.01	0.09	1.55	0.26	0.19	0.012	0.038	0.0037	0.030	60 drops ≈ 1.80 mL ≈ 1.80 g
FS-18	0.90	0.06	0.61	0.07	1.84	0.14	0.14	0.009	0.032	0.0097	0.055	50 drops = 1.35 mL ≈ 1.35 g
FS-19	0.96	0.15	0.48	0.18	1.41	0.23	0.38	0.013	0.040	0.0026	0.100	120 drops ≈ 3.60 mL ≈ 3.60 g
FS-20	1.73	0.16	0.79	0.18	3.12	0.13	0.39	0.026	0.049	0.0081	0.072	125 drops = 3.75 mL ≈ 3.75 g
FS-21	0.57	0.13	0.32	0.15	1.13	0.04	0.32	0.011	0.020	0.0029	0.006	60 drops = 3 mL ≈ 3 g
FS-22	3.08	1.06	0.59	0.08	5.99	0.54	0.30	0.009	1.882	0.0820	0.928	4 capsules = 1.60 g
FS-23	2.59	0.42	1.33	0.49	4.41	0.28	1.05	0.037	0.203	0.0174	0.027	5 pastilles = 10 g
FS-24	3.15	0.53	1.31	0.61	5.43	0.26	1.31	0.045	0.127	0.0088	117.250	5 pastilles = 12.50 g
FS-25	3.80	0.74	1.84	0.86	6.86	0.25	1.84	0.062	0.108	0.0134	0.054	5 pastilles = 17.50 g
FS-26	1.40	0.42	1.12	0.49	4.41	0.84	1.05	0.035	0.141	0.0076	0.028	10 mL ≈ 10 g
FS-27	7.35	1.26	3.36	1.47	17.85	1.05	3.15	0.108	0.292	0.0397	0.391	30 mL ≈ 30 g
FS-28	9.34	1.75	5.25	2.04	21.31	1.75	4.38	0.149	1.025	0.0523	0.278	30 mL = 41.70 g
FS-29	0.52	0.05	0.16	0.05	1.88	0.15	0.12	0.004	0.021	0.0018	0.004	1.1 mL ≈ 1.10 g
FS-30	0.22	0.02	0.10	0.03	0.68	0.06	0.06	0.002	0.005	0.0008	0.003	0.55 mL ≈ 0.55 g
FS-31	0.42	0.05	0.18	0.06	0.92	0.06	0.13	0.005	0.056	0.0010	0.004	1 mL = 1.20 g
FS-32	0.81	0.07	0.45	0.08	1.28	0.13	0.18	0.012	0.031	0.0027	0.016	1.68 mL ≈ 1.68 g
FS-33	0.26	0.02	0.08	0.02	0.34	0.01	0.05	0.002	0.006	0.0004	0.002	0.5 mL ≈ 0.50 g
FS-34	0.84	0.13	1.53	0.15	15.44	1.37	0.46	0.014	0.449	0.0070	0.026	3 tablets = 3 g
FS-35	1.01	0.13	1.62	0.15	13.21	1.47	0.32	0.012	0.439	0.0051	0.052	3 tablets = 3 g
FS-36	1.49	0.15	0.42	0.17	1.72	0.07	0.37	0.012	0.058	0.0028	0.007	5 candies = 3.50 g
FS-37	0.52	0.07	0.27	0.08	2.14	0.09	0.17	0.006	0.033	0.0025	0.012	3 tablets = 1.65 g
FS-38	1.26	0.38	1.07	0.44	4.54	0.19	0.95	0.027	0.055	0.0071	0.021	2 tablets = 9 g
FS-39	0.82	0.19	0.60	0.22	2.11	0.22	0.47	0.016	0.043	0.0043	0.016	45 mL ≈ 45 g
FS-40	4.25	0.95	2.21	1.10	7.88	0.95	2.36	0.078	0.266	0.0304	0.106	22.5 mL ≈ 22.50 g
FS-41	16.60	1.49	7.43	1.73	26.27	1.24	3.72	0.270	0.567	0.0513	0.258	30 mL = 35.40 g
FS-42	3.68	0.63	2.21	0.74	9.14	0.63	1.58	0.058	0.135	0.0170	0.064	6 pastilles = 15 g
FS-43	7.56	1.26	4.20	1.47	18.48	2.52	3.15	0.108	0.355	0.0525	0.403	30 mL ≈ 30 g
FS-44	3.29	0.42	1.40	0.49	7.63	1.68	1.05	0.039	19.250	0.0257	0.078	10 mL ≈ 10 g
FS-45	1.68	0.42	1.40	0.49	3.78	0.14	1.05	0.040	0.047	0.0066	0.014	10 mL ≈ 10 g
FS-46	5.78	0.63	3.26	0.74	10.61	0.63	1.58	0.108	0.143	0.0203	0.080	3 teaspoons = 15 g
FS-47	4.62	0.63	3.57	0.74	10.92	0.63	1.58	0.099	0.131	0.0194	0.070	15 mL ≈ 15 g
FS-48	13.42	1.37	6.14	1.59	27.53	2.05	3.64	0.221	0.494	0.0644	0.457	5 teaspoons = 32.50 g
FS-49	6.51	0.63	3.89	0.74	13.65	1.05	2.10	0.107	0.296	0.0312	0.446	15 g
FS-50	8.93	1.05	1.79	0.74	20.69	1.68	1.58	0.059	0.647	0.1691	0.765	15 g
FS-51	6.72	0.63	3.15	0.74	25.52	2.63	1.58	0.060	0.844	0.2688	0.872	15 g

** For samples with weight not indicated within the recommended daily dose in units of mass; 1 mL corresponds to 1 g, for drops with the amount not indicated in units of volume, 1 drop matches to 0.03 mL, which corresponds to 0.03 g.

**Table 5:** Criteria of the toxic elements’ limits [mg/kg bw/week] calculated for person of 70 kg body weight (bw) per week.

Criteria	Pb (Total)	Cd	As (Inorganic Derivate)	Hg (Inorganic)	Ni	Co (Total)	Se	Cr (VI)	Fe	Cu	Zn
BMDL01	0.245		0.3381								
NOAEL				112.7							
BMDL10								53.9			
BMDL05								98			
LOAEL						490					
DNEL		0.49			5.39		2.107		347.9	20.09	406.7

BMDL: Benchmark Dose Lower Confidence Limit; NOAEL: No Observed Adverse Effect Level; LOAEL: Lowest Observed Adverse Effect Level; DNEL: Derived No-Effect Level.

**Table 6:** Ranking of the observed samples.

Sample Code	SS	BMDL01	NOAEL	BMDL10	BMDL05	LOAEL	DNEL
FS-01	0.487	0.380	1.000	0.990	0.995	1.000	0.692
FS-02	0.513	0.292	1.000	0.990	0.995	1.000	0.699
FS-03	0.476	0.300	1.000	0.993	0.996	1.000	0.784
FS-04	**0.658**	0.380	1.000	0.983	0.990	1.000	0.471
FS-05	0.487	0.395	0.999	0.994	0.997	1.000	0.622
FS-06	0.545	0.970	**1.000**	**1.000**	**1.000**	**1.000**	0.824
FS-07	0.537	0.832	1.000	0.999	0.999	1.000	0.814
FS-08	0.518	0.551	1.000	0.994	0.997	1.000	0.804
FS-09	0.532	0.841	1.000	0.998	0.999	1.000	0.811
FS-10	0.315	0.175	0.999	0.981	0.990	1.000	0.551
FS-11	0.465	0.365	1.000	0.990	0.995	1.000	0.806
FS-12	0.516	0.339	1.000	0.980	0.989	1.000	0.625
FS-13	0.317	0.241	0.999	0.906	0.948	0.992	0.549
FS-14	0.357	0.102	0.999	0.881	0.934	1.000	0.725
FS-15	0.392	0.241	1.000	0.913	0.952	1.000	0.698
FS-16	0.534	0.900	1.000	0.989	0.994	1.000	0.823
FS-17	0.523	0.812	1.000	0.985	0.992	1.000	0.825
FS-18	0.531	0.837	1.000	0.989	0.994	1.000	0.821
FS-19	0.521	0.841	1.000	0.984	0.991	1.000	0.823
FS-20	0.511	0.692	1.000	0.967	0.982	1.000	0.816
FS-21	0.527	0.917	1.000	0.987	0.993	1.000	0.827
FS-22	0.482	0.507	1.000	0.989	0.994	1.000	0.659
FS-23	0.473	0.504	1.000	0.953	0.974	1.000	0.798
FS-24	0.549	0.421	1.000	0.943	0.968	1.000	0.634
FS-25	0.424	0.312	0.999	0.920	0.956	1.000	0.789
FS-26	0.480	0.707	1.000	0.955	0.975	1.000	0.805
FS-27	0.340	0.153	0.999	0.861	0.923	1.000	0.722
FS-28	0.249	0.000	0.999	0.807	0.894	1.000	0.674
FS-29	0.541	0.942	1.000	0.995	0.997	1.000	0.828
FS-30	0.545	**0.994**	1.000	0.998	0.999	1.000	0.832
FS-31	0.539	0.955	1.000	0.994	0.997	1.000	0.829
FS-32	0.530	0.867	1.000	0.986	0.992	1.000	0.827
FS-33	0.544	0.990	1.000	0.998	0.999	**1.000**	**0.833**
FS-34	0.534	0.750	1.000	0.983	0.990	1.000	0.778
FS-35	0.528	0.715	1.000	0.986	0.992	1.000	0.784
FS-36	0.520	0.767	1.000	0.985	0.992	1.000	0.824
FS-37	0.537	0.930	1.000	0.993	0.996	1.000	0.826
FS-38	0.490	0.734	1.000	0.966	0.981	1.000	0.809
FS-39	0.516	0.850	1.000	0.980	0.989	1.000	0.821
FS-40	0.392	0.273	0.999	0.900	0.945	1.000	0.765
FS-41	0.184	0.000	0.999	0.650	0.807	1.000	0.686
FS-42	0.436	0.273	1.000	0.925	0.959	1.000	0.784
FS-43	0.328	0.066	0.999	0.860	0.923	1.000	0.711
FS-44	0.488	0.390	1.000	0.950	0.972	1.000	0.624
FS-45	0.474	0.635	1.000	0.949	0.972	1.000	0.811
FS-46	0.399	0.164	1.000	0.860	0.923	1.000	0.778
FS-47	0.405	0.132	1.000	0.872	0.930	1.000	0.778
FS-48	0.246	0.000	0.999	0.714	0.843	1.000	0.675
FS-49	0.395	0.098	1.000	0.862	0.924	1.000	0.749
FS-50	0.423	0.317	1.000	0.924	0.958	1.000	0.623
FS-51	0.451	0.175	1.000	0.923	0.958	1.000	0.555

BMDL: Benchmark Dose Lower Confidence Limit; NOAEL: No Observed Adverse Effect Level; LOAEL: Lowest Observed Adverse Effect Level; DNEL: Derived No-Effect Level. Bolded values belong to the samples ranked the best according to the observed criteria: Sample FS-04 was given the best score according to the Standard Scores (SS) criterion; Sample FS-06 was given the best score according to the four criteria: NOAEL, BMDL10, BMDL05, and LOAEL; Sample FS-30 was given the best score according to the two criteria: LOAEL, DNEL.

## Data Availability

The data presented in this study are available on request from the corresponding author. The data are not publicly available due to privacy.

## Supplementary Materials

The following supporting information can be downloaded at: https://www.mdpi.com/article/10.3390/foods11091279/s1, Table S1: The recording conditions on ICP-MS; Table S2: Detection limits (LOD) and quantification limits (LOQ) for tested toxic metals and micronutrients; Table S3a: List of certified reference materials; Table S3b: List of chemicals and reagents; Table S4: List of toxic element criteria by different bibliographic sources.
