# Supplementary material for: Assessment of Metal Intake by Selected Food Supplements Based on Beehive Products
Source: Foods. 2022 Apr 28;11(9):1279. doi: 10.3390/foods11091279 (PMC9101606; doi:10.3390/foods11091279)
Supplement: Supplementary file 1 [file foods-11-01279-s001.zip › foods-1656024-SI.pdf]

Table S1. The recording conditions on ICP-MS

Parameter	Recording condition
Atomizer	MicroMist
Injector	quartz
Cones	nickel
RF power	1180 W
Plasma gas flow	15.0 L/min
Atomizer gas flow	1.07 L/min
Auxiliary gas flow	0.90 L/min
Integration time	1000 ms
Spot points	100
Number of replicas	5
Delay time	30 s
Rinse time	70 s

Table S2. Detection limits (LOD) and quantification limits (LOQ) for tested toxic metals and micronutrients

Element	LOD [mg/kg]	LOQ [mg/kg]
Lead	0.005	0.020
Cadmium	0.002	0.006
Arsenic	0.004	0.014
Mercury	0.002	0.007
Chromium	0.015	0.050
Iron	0.15	0.500
Cobalt	0.0006	0.002
Nickel	0.02	0.050
Copper	0.02	0.070
Zinc	0.03	0.100
Selenium	0.004	0.015

Table S3a. List of certified reference materials

Name	Purity	Manufacturer
Cobalt (Co)	1002.2±4.6 mg/L	CPAchem Ltd., Bulgaria
Copper (Cu)	1000±2 mg/L	Reagecon Diagnostics Ltd., Ireland
Zinc (Zn)	995.5±5 mg/L	CPAchem Ltd., Bulgaria
Cadmium (Cd)	999.3±5.1 mg/L	CPAchem Ltd., Bulgaria
Nickel (Ni)	1009±3 mg/L	LGC, USA
Chromium (Cr)	998.5±3.1 mg/L	CPAchem Ltd., Bulgaria
Lead (Pb)	1000±2 mg/L	Reagecon Diagnostics Ltd., Ireland
Selenium (Se)	1001±2 mg/L	Reagecon Diagnostics Ltd., Ireland
Iron (Fe)	1000.3±5.8 mg/L	CPAchem Ltd., Bulgaria
Mercury (Hg)	995±5 mg/L	Fisher Chemical, USA
Arsenic (As)	1001.7±7.2 mg/L	CPAchem Ltd., Bulgaria

Table S3b. List of chemicals and reagents

Name	Purity	Manufacturer
Potassium bromide (KBr)	p.a.	ITRIJ d.o.o., Slovenia
Potassium bromate ($KBrO_3$)	p.a.	ITRIJ d.o.o., Slovenia
Nitric acid (HNO_3)	≥ 65% w/w	Scharlab, S.L., Spain
Sodium hydroxide (NaOH)	p.a.	Merck, Germany
Hydrochloric acid (HCl)	37%	Scharlab, S.L., Spain
Internal standard (mix In, Bi, Rh, Sc)	≥ 99.99%	Agilent, USA
Argon (Ar)	6.0	Messer, Austria
Helium (He)	6.0	Messer, Austria
Hydrogen peroxide (H_2O_2)	30%	Alkaloid Skopje, Republic of North Macedonia

Table S4. List of toxic element criteria by different bibliographic sources

Substance	Author	Year	Study	Route	Endpoint	Qualifier	Value	Unit	Effect	Toxicity
*Pb (total)	EFSA CONTAM	2010	Human health	Oral: unspecified	BMDL01	=	0.5	µg/kg bw/day	Neurology	Developmental
*As (inorganic derivate)	EFSA CONTAM	2009	Human health	Not reported	BMDL01	<=	0.69	µg/kg bw/day	Histopathology neoplastic	Pulmonary and cardiac
*Hg (inorganic)	EFSA CONTAM	2008	Human health	Not reported	NOAEL	=	0.23	mg/kg bw/day	Not reported	Nephrotoxicity
*Chromium (VI)	EFSA CONTAM	2014	Human health	Oral: drinking water	BMDL10	=	0.11	mg/kg bw/day	Histopathology non neoplastic	---
*Chromium (VI)	EFSA CONTAM	2014	Human health	Oral: drinking water	BMDL05	=	0.2	mg/kg bw/day	Haematology	Hemopoietic
*Cobalt (total)	EFSA FEEDAP	2012	Human health	Oral: unspecified	LOAEL	ca.	1	mg/kg bw/day	Haematology	Hemopoietic
**Cadmium	ECHA	2021	Human health	Oral	DNEL	=	1	µg/kg bw/day	---	---
**Nickel	ECHA	2021	Human health	Oral	DNEL	=	0.011	mg/kg bw/day	---	---
**Copper	ECHA	2021	Human health	Oral	DNEL	=	0.041	mg/kg bw/day	---	---
**Zinc	ECHA	2021	Human health	Oral	DNEL	=	0.83	mg/kg bw/day	---	---
**Selenium	ECHA	2021	Human health	Oral	DNEL	=	4.3	µg/kg bw/day	---	---
**Iron	ECHA	2021	Human health	Oral	DNEL	=	0.71	µg/kg bw/day	---	---

EFSA CONTAM: European Food Safety Agency, the Panel on Contaminants in the Food Chain; EFSA FEEDAP: European Food Safety Agency, the Panel on Additives and Products or Substances used in Animal Feed.
*Zenodo. OpenFoodTox: EFSA's chemical hazards database [23];
**Cadmium, nickel, copper, zinc, selenium, iron [24-29].
